# Hydrogels incorporating active compounds from traditional Chinese medicine for diabetic wound healing: mechanistic pathways and bioengineering progress

**DOI:** 10.3389/fcell.2025.1666646

**Published:** 2025-09-09

**Authors:** Rui Zhang, Suk Fei Tan, Ye Wang, Junxue Wu, Chao Zhang

**Affiliations:** ^1^ Department of Orthopaedics, Affiliated Hospital of North Sichuan Medical College, Nanchong, Sichuan, China; ^2^ School of Graduate Studies, Post Graduate Centre, Management and Science University, Shah Alam, Malaysia; ^3^ School of Pharmacy, Management and Science University, Shah Alam, Malaysia

**Keywords:** diabetic wound healing, hydrogels, traditional Chinese medicine, mechanisms, stimuli-responsive, self-assembly, bioactive dressings

## Abstract

Diabetic wounds, especially foot ulcers, pose significant clinical challenges due to persistent inflammation, oxidative stress, impaired angiogenesis, and a high risk of infection. Advanced therapeutic strategies are needed to actively modulate the wound microenvironment. Hydrogels incorporating bioactive compounds derived from Traditional Chinese Medicine (TCM), such as curcumin, baicalein, glycyrrhetinic acid, Astragalus polysaccharides, and Ganoderma lucidum polysaccharides, offer a promising integrative approach. These hydrogels combine the biological activities of TCM compounds with the advantages of a moist, biocompatible wound dressing. This review highlights recent advancement (2020–2025) in TCM-based hydrogels for diabetic wound healing focusing on the design of these materials (e.g., curcumin, baicalein, glycyrrhetinic acid, Astragalus and Ganoderma polysaccharides) and the development of stimuli-responsive delivery systems (e.g., pH, enzymes, temperature, glucose and possibly magnetic/electric fields). TCM-derived compounds can not only form or reinforce hydrogel networks but also impart therapeutic functions by modulating key cellular pathways involved in anti-inflammatory (NF-κB) and antioxidant responses (Nrf2/HO-1), angiogenesis (VEGF, PI3K/Akt), and tissue regeneration (TGF-β/Smad). Challenges in translating TCM-based hydrogles into clinical use, such as pharmacokinetic variability and stability of the active compounds, are also discussed. Furthermore, representative studies are critically compared to elucidate how different TCM–hydrogel systems enchance wound healing outcomes by improving tissue regeneration, accelerating wound closure, and combating infection through responsive release and localized delivery mechanism. TCM-based hydrogels offer a novel, multi-functional platforms to diabetic wounds. They represent a novel paradigm in chronic wound management. Continued interdisciplinary research and clinical translation of these integrative biomaterials could significantly advance precision regenerative therapy for diabetic patients.

## 1 Introduction

Diabetic wounds, especially diabetic foot ulcers, are chronic injuries characterized by prolonged inflammation, impaired cell proliferation, reduced angiogenesis, and a high risk of infection. These factors lead to slow or non-healing wounds that significantly burden patients and healthcare systems (Mees et al., 2023). Globally, chronic wounds affect up to 1%–2% of the population, and diabetic foot ulcers have amputation rates as high as 14%–24% (Yang et al., 2024c). The hyperglycemic environment in diabetic patients causes neuropathy and vascular dysfunction, resulting in poor wound healing outcomes (Armstrong et al., 2023; Reardon et al., 2020). Current clinical treatments (e.g., debridement, pressure off-loading, and standard dressings) often fail to effectively resolve the complex pathology of diabetic wounds (Ur Rehman et al., 2023). Therefore, innovative wound dressings that can actively modulate the wound microenvironment are urgently needed.

Hydrogels have attracted intense interest as wound dressings for diabetic ulcers due to their unique physicochemical properties. Hydrogels are hydrophilic 3D polymer networks that can absorb and retain large amounts of water, providing a moist wound environment that is conducive to healing (Markovic et al., 2024; Zheng et al., 2024; Zhu et al., 2024). Unlike dry gauze, hydrogel dressings keep the wound bed hydrated, which protects tissue, promotes re-epithelialization, and reduces pain. They also conform to wound contours and allow gas exchange. Importantly, hydrogels can be engineered to incorporate bioactive agents (drugs, growth factors, antimicrobials, etc.) and release them in a controlled manner, thus combining physical protection with therapeutic delivery (Gao et al., 2021; Gounden and Singh, 2024). For diabetic foot ulcers, a meta-analysis showed that hydrogel dressings achieved higher healing rates and faster ulcer closure compared to conventional dressings (Zhao et al., 2024a). The biocompatibility and tunable structure of hydrogels make them ideal carriers for advanced wound therapies in diabetes.

Traditional Chinese Medicine (TCM) has long been used to treat chronic wounds using herbal extracts or formulations that possess anti-inflammatory, antioxidant, antimicrobial, and circulation-promoting activities (Ning et al., 2022). Specific compounds from TCM like baicalin (from *Scutellaria baicalensis*) have been shown to accelerate wound closure (Mao et al., 2021; Zhu et al., 2024) and curcumin and glycyrrhetinic acid (Yuan et al., 2025), and many TCM herbs historically were applied topically to treat skin ulcers and sores (Wang et al., 2022; Chanu et al., 2023). TCM’s multi-component, multi-target mechanisms offer a holistic therapeutic effect that could complement modern wound care (Zhou et al., 2024). However, direct application of herbal extracts may be limited by short retention time on the wound, inconsistent composition, or suboptimal dosing.

Integrating TCM components into hydrogels provides a promising strategy to harness the benefits of both traditional medicine and modern biomaterials. Hydrogels can serve as a sustained delivery system and protective matrix for TCM-derived bioactive molecules, improving their stability and localized effect. Meanwhile, the incorporated TCM ingredients impart biological functions to the hydrogel, potentially transforming an inert dressing into a bioactive therapeutic. Such TCM-infused hydrogels can actively modulate the diabetic wound microenvironment–for instance, by releasing anti-inflammatory phytochemicals to resolve chronic inflammation, scavenging excess reactive oxygen species (ROS) through antioxidant herbs, or exerting antibacterial effects to control infection. Additionally, some natural polymers and small molecules from TCM can themselves form or strengthen hydrogel networks, reducing the need for synthetic polymers or harsh crosslinkers (Mees et al., 2023; Zou et al., 2023). This synergy aligns with the concept of “integrative medicine”, combining traditional remedies with advanced drug delivery for improved outcomes. Stimuli-responsive hydrogels, responsive to factors like pH, temperature, glucose, or even magnetic/electric fields, further enhance the therapeutic potential of TCM-based systems (Garshasbi et al., 2024).

Recent advances in TCM-derived hydrogels for diabetic wound healing are reviewed in the following sections. Initially, key TCM components utilized in hydrogel formulations are categorized, with emphasis on their specific contributions to the hydrogel’s structural integrity, such as acting as cross-linkers, network builders, or functional fillers. Subsequently, innovative design strategies—including stimuli-responsive release systems and self-assembling hydrogels that utilize TCM molecules as both structural matrices and therapeutic agents—are examined. A critical comparison of representative studies published between 2020 and 2025 is then presented, evaluating the relative performance of various TCM–hydrogel systems and identifying prevailing trends or existing research gaps. This comparison is followed by an in-depth mechanistic analysis exploring how these TCM components enhance diabetic wound healing at the molecular level, particularly through modulation of critical signaling pathways such as inflammatory cascades and regenerative processes. Integrating traditional medicinal knowledge with advanced biomaterials technology, TCM-derived hydrogels represent a novel, multidimensional approach to addressing the persistent clinical challenges associated with diabetic wound management.

## 2 Role of TCM components in hydrogel network formation

**FIGURE 1:**
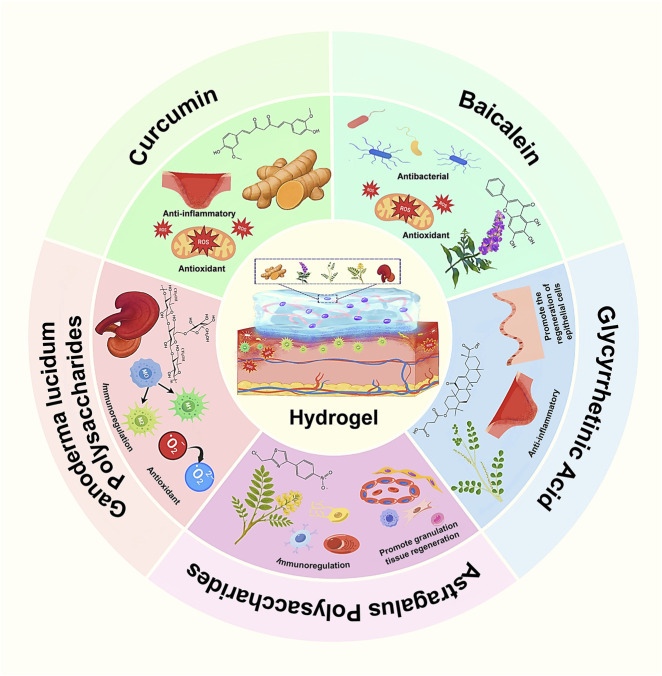
Bioactive traditional Chinese medicine (TCM) ingredients incorporated into a hydrogel scaffold for wound healing. A schematic wheel highlights five representative TCM components—Curcumin, Baicalein, Glycyrrhetinic acid, Ganoderma lucidum polysaccharides, and Astragalus polysaccharides—each linked to its principal biological activity (anti-inflammatory, antioxidant, antibacterial, immunomodulatory, and pro-granulation, respectively).

One of the distinctive advantages of incorporating TCM ingredients into hydrogels is that many of these natural compounds can actively participate in the hydrogel’s network formation. Instead of being passive “cargo”, certain TCM-derived molecules contribute to the cross-linking or structuring of the gel matrix, thus improving the mechanical properties and stability of the hydrogel while also endowing bioactivity (Xiang et al., 2020; Zhai et al., 2024; Li et al., 2025a). This section discusses how specific TCM components–including small molecule polyphenols, triterpenoids, and polysaccharides–influence the formation of hydrogel scaffolds. Key examples include curcumin, baicalein, 18β-glycyrrhetinic acid, Astragalus polysaccharides, and Ganoderma lucidum polysaccharides, among others (Figure 1). These substances can form physical or chemical cross-links through various interactions (hydrogen bonding, π–π stacking, Schiff-base formation, ionic interactions, etc.), resulting in composite hydrogels with enhanced strength, injectability, and therapeutic functionality.

### 2.1 Curcumin

Curcumin, a hydrophobic polyphenol derived from Curcuma longa, is well known for its anti-inflammatory and antioxidant properties and has been extensively studied for its role in wound healing (Akbik et al., 2014; Tejada et al., 2016; Chandio et al., 2022). In hydrogels, curcumin can serve dual roles: as a therapeutic agent and as a network modifier. Curcumin’s structure contains multiple hydroxyl and aromatic groups, which enables it to engage in hydrogen bonding and hydrophobic interactions with polymer networks (Samanta and Roccatano, 2013; Wegiel et al., 2014; Gao et al., 2021). For instance, curcumin has been incorporated into chitosan-based hydrogels where it interacts with polymer chains, thereby slightly increasing gel stiffness and stability while scavenging free radicals in the wound (Chopra et al., 2023). In one study, a thermal-responsive hydrogel composed of chitosan and Pluronic F127 was loaded with curcumin; the curcumin not only provided antioxidant activity but also influenced the gel’s swelling and release behavior (Sideek et al., 2022). The presence of gelatin in a dual-polymer system further densified the network via additional hydrogen bonds, resulting in a more sustained release of curcumin and improved mechanical robustness (Pham et al., 2019) Curcumin can also act as a natural photoinitiator for polymerization under light (Zhao et al., 2015). Enabling *in situ* hydrogel formation without toxic initiators. Thus, curcumin contributes to hydrogel formation by physical cross-linking and network reinforcement, while its inherent bioactivity promotes wound healing (e.g., reducing oxidative stress and inflammation).

### 2.2 Baicalein and Baicalin

Baicalein and Baicalin–flavonoids derived from *Scutellaria baicalensis* (Chinese skullcap) – similarly enhance hydrogel networks. Baicalein (the aglycone) and baicalin (its glycoside) have multiple phenolic hydroxyls that can form hydrogen bonds with polymers such as collagen, gelatin, or chitosan (Liu et al., 2020; Baviskar and Lodhi, 2024). When baicalein was loaded into a glycol chitosan/gellan gum hydrogel, it interacted with the polymer matrix, which improved the gel’s structural integrity and facilitated uniform drug distribution (Baviskar and Lodhi, 2023). These interactions likely contributed to the hydrogel’s ability to maintain its form during application, as well as modulating the release profile of baicalein. In addition, baicalein’s poor water solubility is mitigated by the hydrogel environment, which can prolong its retention at the wound site. Chemically, baicalin (the glycoside form) could be oxidized to generate aldehyde groups (via its sugar moiety) that form Schiff-base cross-links with amino polymers, as done with other polysaccharides (Zhu et al., 2024). Although not yet commonly reported, it is conceivable that baicalein could be grafted onto polymer backbones to serve as a cross-linking node, similar to how gallic acid (a tri-phenolic acid) has been grafted onto gelatin to create antioxidant hydrogel networks (Zhang et al., 2016). The inclusion of baicalein/baicalin in hydrogels not only reinforces the network through additional bonding but also introduces strong antioxidant and antibacterial functions that are beneficial for diabetic wounds (Manconi et al., 2018; Liu et al., 2020; Baviskar and Lodhi, 2023; Nie et al., 2023). These flavonoids help quench excess ROS and inhibit microbial growth, thereby protecting cells in the wound. Indeed, hydrogels containing baicalein have demonstrated markedly enhanced fibroblast proliferation and migration in diabetic conditions, partly due to the stabilized presentation of baicalein within the gel matrix.

### 2.3 Glycyrrhetinic acid and glycyrrhizin

Glycyrrhetinic Acid and Glycyrrhizin–triterpenoid compounds from licorice (Glycyrrhiza uralensis) – can play a significant role in hydrogel formation owing to their amphiphilic structures (Özdemir et al., 2022). 18β-glycyrrhetinic acid (GA) is a pentacyclic triterpene aglycone, while glycyrrhizin is GA conjugated with a disaccharide (Li et al., 2016a). Glycyrrhizin, being more water-soluble, has been found to self-assemble into hydrogels when simply dissolved at sufficiently high concentration in water (Mees et al., 2023). The molecule’s hydrophobic triterpenoid backbone and hydrophilic sugar moieties enable it to form a physical cross-linked network through hydrophobic interactions and hydrogen bonding. Specifically, multiple glycyrrhetinic acid units aggregate to form hydrophobic domains (as observed in lattice models), while the hydrophilic parts maintain a water-swollen network (Izutani et al., 2014; Petrova et al., 2017). This yields a physical hydrogel without any synthetic polymer–a phenomenon exploited to create glycyrrhizin-based wound dressings. Such hydrogels can have “tunable” rheological properties by simply adjusting glycyrrhizin concentration (Mees et al., 2023). On the other hand, despite its poor water solubility, GA can form carrier-free hydrogels and nanodispersions through self-assembly or co-assembly with amphiphilic derivatives like diammonium glycyrrhizinate (Cheng et al., 2022; Zhao et al., 2024b). These self-assembled structures enhance GA’s solubility, stability, and bioavailability, making it a promising candidate for drug delivery systems. A recent study prepared a *carrier-free* hydrogel by heating and cooling glycyrrhetinic acid in water, without any additional cross-linker (Zou et al., 2023) The heating step presumably dissolved GA (perhaps with transient micellization), and upon cooling, GA molecules self-assembled into a gel network stabilized by non-covalent interactions. This GA hydrogel exhibited good mechanical integrity and leveraged GA’s anti-inflammatory property to improve healing outcomes. Beyond self-gelation, glycyrrhetinic acid can also be conjugated onto polymers. Recent studies have explored the potential of glycyrrhetinic acid (GA) conjugation with polymers to create robust and biologically active hydrogels. GA has been used as a guest molecule for β-cyclodextrin to form inclusion complexes, resulting in self-healing supramolecular polymer hydrogels with excellent biocompatibility (Li et al., 2016a). GA grafted hyaluronic acid or chitosan could cross-link via hydrophobic association of the GA groups, forming hydrogels that are more robust and biologically active (by presenting GA on the matrix) (Li et al., 2023a). In summary, licorice triterpenoids contribute to hydrogel formation either by self-assembling into supramolecular gels or by serving as hydrophobic cross-linking domains when attached to polymers (Özdemir et al., 2022). They also carry bioactivities like HMGB1-inhibition (glycyrrhizin is a known alarmin inhibitor) which can modulate inflammation in wounds.

### 2.4 Astragalus polysaccharides (APS)

Astragalus Polysaccharides (APS) – high-molecular-weight polysaccharides from Astragalus membranaceus–are natural polymers that can be integrated as structural components of hydrogels. PS consist primarily of monosaccharides like rhamnose, xylose, glucose, and galactose, with molecular weights ranging from 120 to 300 kDa (Xu et al., 2008; Fu et al., 2013). The main structural components include rhamnogalacturonan II pectins and α-(1→4)-glucan, with variations in methyl ester levels and molecular weight profiles depending on the plant’s origin (Sheng et al., 2021). APS exhibit numerous biological activities, including immunomodulation, antioxidant, antitumor, anti-diabetic, antiviral, and neuroprotective effects (Jin et al., 2014). APS typically consists of heteropolysaccharide chains with multiple functional groups (hydroxyls, occasionally uronic acids) that make them amenable to cross-linking. One approach to incorporate Astragalus polysaccharides is to oxidize them to generate aldehyde groups (creating oxidized APS, or OAPS), which can then chemically cross-link with amino-containing polymers via Schiff-base (imine) bonds. For instance, Li et al. (2025b) discovered through their research that an APS-based hydrogel can be formed by cross-linking oxidized Astragalus polysaccharide with carboxymethyl chitosan, yielding a biocompatible network. The APS provided a natural backbone, while the imine linkages imparted dynamic covalent characteristics. The APS provided a natural backbone, while the imine linkages imparted dynamic covalent characteristics. Astragalus polysaccharides can also engage in ionic cross-linking: e.g., mixing with alginate (which can be ionically cross-linked by Ca^2+^). In a recent design, a tri-component hydrogel was made from APS, carboxymethyl chitosan (CMCS), and sodium alginate (SA) – APS likely hydrogen-bonded or intertwined with the other polymers, and the whole network was strengthened by Ca^2+^ ions bridging alginate chains (Wen et al., 2023). Including APS in hydrogels often improves the gel’s viscoelasticity and moisture retention due to the high molecular weight and water-binding capacity of polysaccharides. Moreover, APS themselves have immunomodulatory activity (e.g., promoting M2 macrophage polarization and fibroblast activation) that can benefit wound repair (Liu et al., 2011; Zheng et al., 2023). By serving as a functional filler or second network, Astragalus polysaccharides reinforce the hydrogel matrix and provide a reservoir of bioactive sugar chains that are gradually released or presented to cells. This dual role is exemplified by studies where APS-containing hydrogels showed enhanced tensile strength and stretchability (Wen et al., 2023). Simultaneously, APS acts as a bioactive component, accelerating wound closure through anti-inflammatory, procollagen deposition, and proangiogenic effects (Wu et al., 2022b; Wen et al., 2023). This two-pronged approach combines biomechanical and biochemical functionalities to promote wound healing (Li et al., 2020b). APS-containing hydrogels demonstrate superior wound healing capabilities compared to other nanocomposite gels, showing increased re-epithelialization, hair follicle regeneration, and improved α-SMA expression (Youssef et al., 2024). The gradual release of APS from the hydrogel matrix provides a sustained therapeutic effect, making it an effective approach for joint wound care and soft tissue management (Wu et al., 2022b; Wen et al., 2023). These findings underscore the potential of APS-based hydrogels in advanced wound healing applications.

### 2.5 Ganoderma lucidum polysaccharides (GLP)

Ganoderma lucidum Polysaccharides (GLP) – polysaccharides extracted from the medicinal fungus Ganoderma lucidum (Reishi mushroom) – similarly can act as both scaffold materials and active agents. GLPs are branched β-glucans and heteroglycans known for immunomodulatory and antioxidant effects (Li et al., 2020a; Lu et al., 2020). These polysaccharides exhibit antioxidant, immunomodulatory, anti-inflammatory, antitumor, and antidiabetic activities (Seweryn et al., 2021). These polysaccharides can be chemically modified (e.g., oxidized to form aldehydes) to directly cross-link hydrogels. According to the research by Li et al. (2024a), oxidized Ganoderma lucidum polysaccharide (OGLP) was cross-linked with carboxymethyl chitosan and alginate to form a double-network hydrogel, which demonstrated significant effects in accelerating diabetic wound healing. In this system, OGLP provided one network via Schiff-base bonds with chitosan, while alginate formed another via ionic gelation–resulting in a sturdy gel capable of adhering to tissue. The GLP-based network significantly improved the hydrogel’s mechanical properties (e.g., higher compression modulus) and tissue adhesion. The multitude of hydroxyl groups on GLP can also form hydrogen bond cross-links in physically cross-linked gels (for instance, blending GLP with polyvinyl alcohol followed by freeze-thaw cycling could yield interpenetrating networks). Importantly, Ganoderma polysaccharides imbue hydrogels with bioactivity: they have been shown to scavenge ROS and modulate immune cells (Zhao et al., 2020a; Li et al., 2024a). As part of a hydrogel, GLP continuously released can shift macrophages from a pro-inflammatory (M1) state to a pro-healing (M2) state within the wound, thereby accelerating the resolution of inflammation (Li et al., 2024a). In a diabetic wound context, such GLP-containing hydrogels not only physically fill the wound and maintain moisture, but also actively encourage a regenerative microenvironment through their biochemical signals. Therefore, incorporating Ganoderma lucidum polysaccharides into hydrogels serves a tri-fold purpose: forming a robust matrix, providing controlled release of an active polysaccharide, and enhancing healing via immune regulation and antioxidation.

In summary, TCM components can significantly influence hydrogel network formation. Small molecule compounds like curcumin and baicalein often engage in non-covalent interactions that fortify the polymer mesh, whereas natural macromolecules like triterpenoid saponins and polysaccharides can form either supramolecular or chemically cross-linked networks. The result is a range of composite hydrogels wherein the TCM ingredients are integral to the scaffold’s architecture. This integration improves the mechanical stability (preventing premature dissolution and prolonging dressing lifespan) and ensures that bioactive molecules are not rapidly washed away but instead retained in the wound bed. By designing hydrogels where TCM components are part of the backbone or cross-link junctions, researchers have created “bioactive scaffolds” that combine the structural support of a biomaterial with the therapeutic functions of herbal medicine. These hydrogels thus provide a foundation for controlled and effective healing in the challenging milieu of diabetic wounds.

## 3 Controlled release mechanisms of TCM in hydrogels

**FIGURE 2:**
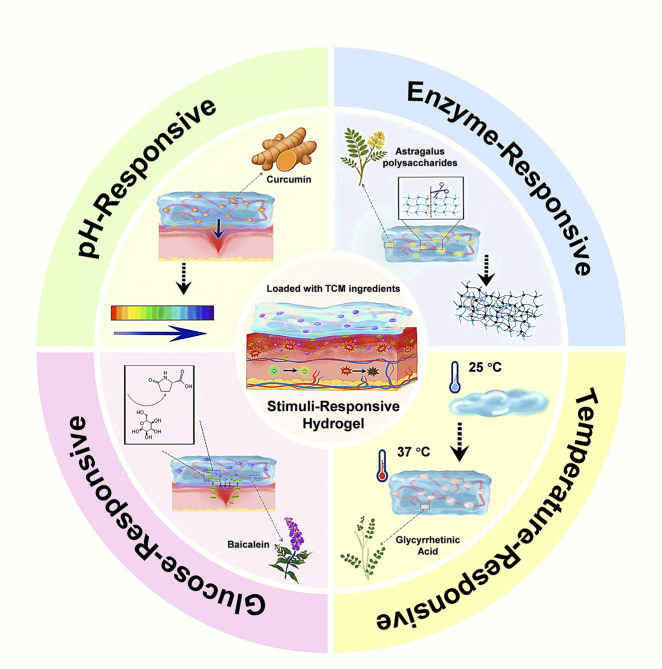
Stimuli-responsive release mechanisms of TCM-loaded hydrogels. The circular diagram is divided into four quadrants showing distinct environmental triggers: pH-responsive (acidic microenvironments induce hydrogel swelling and accelerate curcumin release); enzyme-responsive (matrix-metalloproteinase cleavage of Astragalus polysaccharide cross-links enhances payload diffusion); temperature-responsive (a glycyrrhetinic-acid-containing sol undergoes a sol-to-gel transition as temperature rises from 25 °C to 37 °C); and glucose-responsive (elevated glucose disrupts boronate-ester bonds, liberating baicalein).

A critical aspect of wound dressings that deliver therapeutics is the ability to release those agents in a controlled and timely manner. In diabetic wounds, the microenvironment differs significantly from healthy tissue–it often features abnormal pH, elevated levels of enzymes (such as matrix metalloproteinases), fluctuating temperature in peripheral limbs, and especially high glucose concentrations (Zhao et al., 2024d). Designing hydrogels that respond to these local stimuli can ensure that TCM-derived actives are released “on demand” when and where they are needed. In this section, we discuss how TCM-loaded hydrogels achieve controlled or stimuli-responsive release, focusing on four major triggers: pH, enzymes, temperature, and glucose (Figure 2). Many advanced hydrogel systems combine multiple triggers or a thermo-responsive gel that also responds to enzymes. Representative systems and their release mechanisms are summarized in Supplementary Table 1. An extended overview of controlled release strategies is provided (Supplementary Table 2).

### 3.1 pH-responsive release

Chronic wounds often exhibit a pH that deviates from neutrality. While normal skin is slightly acidic (pH ∼5.5), infected or chronic wounds can become alkaline (pH 7–9) due to bacterial metabolites, or sometimes slightly acidic due to ischemia (Wallace et al., 2019). This alkaline environment can promote bacterial growth, impair healing, and affect various physiological processes including oxygen release, angiogenesis, and protease activity (Jones et al., 2015). PH-responsive hydrogels are intelligent polymer materials that swell, contract, or degrade in response to specific pH conditions (Gao and Xu, 2025). These hydrogels incorporate pH-sensitive linkages or side groups. A common strategy is to use Schiff base (imine) cross-links formed by reactions between aldehyde and amine groups. These dynamic covalent bonds are relatively stable at neutral-to-basic pH but hydrolyze in acidic environments (Psarrou et al., 2023). Thus, a hydrogel crosslinked via imine bonds can remain intact in normal tissue (around pH 7) but gradually break apart and release its payload in a more acidic milieu. For example, oxidized polysaccharides (providing aldehyde groups) crosslinked with chitosan (providing amino groups) form an injectable self-healing hydrogel via imine bonds; when the wound becomes acidic (pH ∼5–6), these bonds cleave, accelerating the release of encapsulated herbal compounds (Liu et al., 2022; Zhou et al., 2023; Jeong et al., 2025). This mechanism is useful in diabetic wounds that turn acidic upon bacterial infection or inflammation–the hydrogel delivers more drug exactly when infection flares. Conversely, hydrogels can also be designed to respond to alkaline pH. Polymers bearing carboxylic acid moieties stay protonated (and form hydrogen-bonded or collapsed networks) at low pH, but deprotonate and ionically repel each other at higher pH, leading to swelling and enhanced drug diffusion. For instance, a chitosan-based hydrogel combined with an anionic polysaccharide (e.g., alginate or carboxymethyl cellulose) will shrink in acidic conditions due to charge neutralization and hydrogen bonding, yet swell in an alkaline environment as carboxylate groups ionize and repel (Kopač et al., 2021; Xiao et al., 2024). Zhao et al. (2023) demonstrated a dual pH/glucose-responsive hydrogel using phenylboronic acid (PBA) chemistry: gelatin and oxidized hyaluronic acid were crosslinked by dynamic boronate ester bonds (forming a network called “GOHA-PBA”) that bound curcumin. At acidic pH, these boronate bonds dissociate, releasing curcumin more rapidly. In essence, by tuning the hydrogel’s cross-link chemistry and ionizable groups, pH-sensitive hydrogels can achieve faster release of TCM actives in environments indicative of infection or chronic inflammation, while retaining the drugs during more quiescent, near-neutral conditions.

### 3.2 Enzyme-responsive release

Diabetic wounds are notorious for an imbalance in proteolytic enzymes, particularly matrix metalloproteinases (MMPs). Chronic wound fluid contains excessively high levels of MMP-9, MMP-2, and other proteases which degrade extracellular matrix components and growth factors, impeding healing (Krishnaswamy et al., 2017; Mullin et al., 2023). Enzyme-responsive hydrogels are designed to leverage these elevated enzymes to trigger drug release *in situ*. One approach is to incorporate peptides or polymer segments that serve as substrates for the target enzymes. When a specific enzyme (e.g., MMP-9) is present at the wound site, it will cleave those substrates, breaking the hydrogel network or a linkage and thereby releasing the encapsulated therapeutic (Chandrawati, 2016; Noddeland et al., 2023). For example, hydrogels crosslinked with gelatin (a denatured collagen peptide) are inherently sensitive to gelatinases like MMP-9. In a high-MMP environment, the gelatin crosslinks or gelatin-based particles degrade enzymatically, causing the hydrogel to soften or disintegrate and liberate its payload. A recent study encapsulated an anti-inflammatory drug (celecoxib) in gelatin microspheres distributed within a chitosan-PBA hydrogel. In the protease-rich environment of a diabetic ulcer, MMP-9 gradually digested the gelatin microspheres, triggering sustained release of celecoxib (Zhou et al., 2022). Notably, this system was dual-responsive: the hydrogel’s boronate cross-links responded to glucose, while the gelatin component responded to MMPs to release the drug–a design that addresses both hyperglycemia and excessive inflammation in wounds. In a similar vein, one could attach TCM compounds to biodegradable linkers that are enzyme cleavable. For instance, curcumin or a TCM polysaccharide might be conjugated to a gel backbone via an MMP-sensitive peptide. Upon MMP cleavage, the bond is broken and the curcumin is freed at the wound site. Apart from MMPs, other wound-associated enzymes can be harnessed. Lysozyme, present in wound exudate, slowly degrades chitosan, thus a chitosan-based hydrogel will gradually erode as lysozyme cleaves its glycosidic bonds (Kim et al., 2020), providing a baseline release of loaded herbal extracts. By matching the hydrogel’s degradability to the enzymatic profile of diabetic wounds, enzyme-responsive hydrogels protect TCM actives during transit and then unload them preferentially in the protease-rich wound bed. This targeted release in response to pathological enzymes helps preserve growth factors and matrix in the wound (by localizing drug action) and times the therapeutic delivery to when it is most needed.

### 3.3 Temperature-responsive release

Temperature-sensitive hydrogels, such as those based on poly (N-isopropylacrylamide) (PNIPAAm) or certain block copolymers (e.g., Pluronic F127, gelatin methacrylate), undergo a sol-gel phase transition near physiological temperature. For wound applications, the typical goal is an injectable liquid at room temperature that gels upon warming to body temperature (∼37 °C) (Zubik et al., 2017; Ávila-Salas and Durán-Lara, 2019). This feature is particularly useful for irregularly shaped or deep wounds, allowing a liquid hydrogel precursor (mixed with TCM actives) to fill the wound and then solidify *in situ*. Thermo-gelling hydrogels can provide a sustained release as the drug diffuses out of the semi-solid gel at body temperature. In some cases, the temperature change itself can modulate release–for instance, gelation might entrap the drug, slowing release, whereas below the gelation temperature the formulation is liquid and might release faster (though in practice, for wound dressings, one generally maintains them at body temperature after application). Sideek et al. (2022) reported that a Pluronic F127–chitosan hydrogel loaded with curcumin remains a low-viscosity, free-flowing solution at room temperature but undergoes a rapid sol–gel transition upon contact with body-temperature tissue, thereby forming a stable *in situ* gel. An advanced design by Zhou et al. (2022) utilized a shape-memory injectable hydrogel that was fluid at low temperature and became viscoelastic at ∼37 °C, helping it adapt to deep wound shapes and then stay firmly in place. Importantly, temperature changes can be combined with other triggers: e.g., a hydrogel might remain liquid for easy application (cooler than body temperature) and then gel at body temperature to serve as a scaffold, while also containing enzyme-cleavable or pH-sensitive bonds that control drug release once the gel is formed. In summary, thermo-responsive hydrogels improve the localization and residence time of TCM therapies in wounds by utilizing the wound’s warmth to trigger gelation. While the temperature itself may not drastically accelerate drug release (once gelled, release is governed by diffusion/degradation), the ability to transition from liquid to solid on contact with the body is crucial for efficient delivery and retention of herbal medicines on the wound site.

### 3.4 Glucose-responsive release

Perhaps the most distinctive feature of diabetic wound fluids is the elevated glucose level in tissue and exudate, which correlates with poor healing outcomes (Spampinato et al., 2020). Glucose-responsive hydrogels aim to link drug release to local glucose concentration so that more therapeutics are released during hyperglycemic episodes or in high-glucose microenvironments. A widely used mechanism involves phenylboronic acid (PBA) or its derivatives, which can reversibly bind diol-containing molecules like glucose. In the absence of glucose, PBA moieties on adjacent polymer chains can form boronate ester cross-links with diol-bearing groups (for example, with poly (vinyl alcohol) or catechol-modified polymers), stabilizing the hydrogel network. When glucose is present at high levels, it competitively binds to PBA, breaking those boronate cross-links and causing the gel to swell or partially dissociate, thereby releasing the encapsulated cargo (Dong et al., 2016; Wu et al., 2022a; Morariu, 2023). In TCM-hydrogel contexts, Zhao et al. (2023) created a gelatin–hyaluronic acid hydrogel with PBA moieties (GOHA-PBA) that could actively “bind” curcumin at high capacity, forming a Cur–PBA complex within the gel. In a high-glucose environment (like a diabetic wound with elevated local glucose), the glucose molecules bind to PBA, displacing the curcumin which then gets released. Additionally, the binding of glucose to PBA increases the hydrogel’s swelling (due to formation of charged boronate), which further facilitates drug diffusion. This system was shown to release curcumin in a glucose-dependent manner and was termed “smart releasing”. Another approach couple’s glucose sensing with enzymatic reactions: for example, a hydrogel loaded with glucose oxidase (GOx) will convert ambient glucose into gluconic acid. The generated acid can locally lower the pH within the hydrogel, which in turn can trigger pH-sensitive bonds to break (linking back to the pH-responsive designs) and release drugs (Kaniewska et al., 2024). A dual-responsive design by Zhou et al. (2022) integrated both PBA chemistry and enzyme-sensitive components: in high-glucose conditions, boronate bonds in the hydrogel were hydrolyzed, quickly releasing insulin that was embedded in the matrix, while concurrently, high MMP-9 levels degraded gelatin microspheres in the gel to release an anti-inflammatory drug. This multi-trigger “smart” hydrogel delivered insulin to help control local glucose toxicity and an herbal anti-inflammatory to modulate the wound inflammation, exemplifying a synergistic approach for diabetic wounds. More advanced systems have even combined three or more stimuli; for instance, Li et al. (2024b) reported a “triple-editing” hydrogel that responds to glucose, reactive oxygen species (ROS), and pH changes in diabetic wounds. For TCM compounds like berberine or tannic acid, which have diol structures, one could imagine a hydrogel where these compounds are actually bound to PBA in the gel; high glucose would then displace them, providing a glucose-triggered release of the TCM compound itself. Overall, glucose-responsive TCM hydrogels—often using boronate-diol interactions—ensure that when local sugar levels spike (a sign of metabolic stress and a factor that can exacerbate infection and inflammation), the hydrogel correspondingly increases the release of its therapeutic payload. This feedback-driven delivery can improve wound outcomes by addressing one of the root causes of impaired healing (hyperglycemia) and by timing the release of herbal drugs to periods of greatest need.

### 3.5 Multi-modal smart hydrogels

It is noteworthy that many cutting-edge hydrogel dressings integrate multiple responsiveness features to tackle the complex diabetic wound environment. A single diabetic ulcer may simultaneously present mildly alkaline pH, elevated MMPs, high glucose, and excess ROS. Smart hydrogel systems are being designed to sense this combination of signals and modulate the release of multiple drugs accordingly (Khattak et al., 2024; Li et al., 2024c; Meng et al., 2025). For TCM components, whose release timing can be crucial (e.g., releasing an angiogenic herb after the infection is controlled, or an antimicrobial right away when bacteria proliferate), these stimulus-responsive designs offer a level of control not achievable with conventional dressings. Ultimately, by aligning the release profile of herbal therapeutics with the dynamic wound environment, pH/enzymatic/thermal/glucose-responsive hydrogels ensure that the diabetic wound receives the right intervention at the right time, thereby optimizing the healing process.

### 3.6 Magnetic/electric field-responsive release

Beyond pH, enzyme, temperature, and glucose stimuli, external physical triggers such as magnetic and electric fields have emerged as promising tools to control drug release from hydrogels (Sun et al., 2020). Magnetic field-responsive hydrogels typically incorporate magnetic nanoparticles (e.g., Fe_3_O_4_), which generate localized heating or mechanical stress under an alternating magnetic field, thereby accelerating drug diffusion or breaking dynamic crosslinks. Similarly, electric field-responsive hydrogels contain conductive polymers or ions that alter hydrogel porosity and electrostatic interactions upon stimulation, enabling on-demand drug release (Liao et al., 2025). These strategies are especially attractive in diabetic wounds with impaired perfusion, where externally applied, non-invasive triggers can precisely modulate the release of TCM-derived actives. Although their clinical application is still limited, the integration of magnetic or electric responsiveness into TCM hydrogels could represent a future direction for enhancing spatiotemporal control of therapeutic delivery (Du et al., 2025).

## 4 Material properties of self-assembled TCM hydrogels

In addition to composite hydrogels that combine polymers and TCM additives, a fascinating area of development is self-assembled hydrogels derived entirely or predominantly from small molecules of TCM origin. These systems rely on the inherent gelation ability of certain natural compounds to form supramolecular hydrogel networks without requiring a polymer scaffold. Such hydrogels are often termed “carrier-free” because the therapeutic itself (or its derivative) becomes the matrix (Hao et al., 2024). They can offer advantages like high drug loading (since the gel is made of the drug or its close analog), biodegradability, and minimal addition of foreign materials. Self-assembly of TCM-derived molecules can create nanodrug delivery systems, improving bioavailability and efficacy of poorly soluble compounds (Li et al., 2024c; Yang et al., 2024b). The material properties, formation mechanisms, and biological functions of self-assembled TCM hydrogels are the focus of this section, with glycyrrhetinic acid (and glycyrrhizin) serving as a key example of triterpenoid-based gelators, along with other small molecules such as mangiferin and puerarin that have demonstrated self-gelation. The organization of these molecules into nanostructures (e.g., fibers, sheets, micelles) forms the basis of the macroscopic gel, accompanied by characteristic mechanical and rheological properties. These hydrogels also exhibit multifunctional roles, including inherent antimicrobial or anti-inflammatory activity and self-healing behavior. Reported dosage and concentration ranges of TCM compounds in hydrogels are listed in Supplementary Table 3.

### 4.1 Formation mechanisms

Self-assembly of small molecules into a hydrogel generally involves a balance of non-covalent forces–hydrogen bonding, π–π stacking, hydrophobic interactions, van der Waals forces, and sometimes coordination bonds (Raeburn et al., 2013; Omar et al., 2022). Traditional Chinese Medicine (TCM) compounds often possess characteristics suitable for self-assembly, including amphiphilic properties and multiple hydrogen bond donors/acceptors (Wang et al., 2012; Li et al., 2024c). For example, glycyrrhizin (the glycosylated form of glycyrrhetinic acid) is amphiphilic, with a hydrophobic triterpene core and hydrophilic sugar chains. In water, glycyrrhizin molecules can aggregate such that the hydrophobic triterpenoid moieties cluster together (minimizing contact with water) while the sugar groups face outward into the aqueous phase (Mees et al., 2023). At high enough concentration, this leads to the formation of a continuous network–glycyrrhizin molecules stack into one-dimensional fibers or two-dimensional sheet-like structures that entangle into a 3D network, physically trapping water and forming a gel. Such physical hydrogels often require a threshold concentration (critical gelation concentration) and sometimes specific triggers (like a pH or temperature change) to initiate ordering (Gao et al., 2015; Petrova et al., 2017; Tucker et al., 2021). In the case of glycyrrhizin, simply dissolving it in water at sufficient concentration (e.g., several percent weight/volume) and allowing the system to equilibrate can produce a gel, as reported by Mees et al. (2023). They observed remarkable rheological properties for glycyrrhizin hydrogels, indicating a well-developed network (Cai et al., 2023). For glycyrrhetinic acid (GA) without the sugar, direct solubilization is challenging; however, as mentioned earlier, heating followed by cooling can drive GA molecules to assemble, likely by first dissolving or forming micelles at high temperature then nucleating into crystalline or fibrous aggregates upon cooling (Zou et al., 2023). The result is a hydrogel where GA molecules are held together by a combination of hydrogen bonds (GA has carboxyl and hydroxyl that can bond) and hydrophobic interactions between the pentacyclic rings. Other small molecules: Mangiferin (a glucoside of a xanthone) is a polyphenol that was recently shown to form a hydrogel upon heating-cooling in water (Hao et al., 2024). The heating step likely helps dissolve or randomize the mangiferin, while cooling allows these interactions to dominate and the ordered aggregates (nanofibers) to form. Similarly, puerarin (a daidzein glycoside from kudzu root) has been investigated for self-assembly: a study in 2025 demonstrated that puerarin and glycyrrhizic acid together can co-assemble into a hydrogel, indicating multiple components can synergize in self-assembly (Ji et al., 2025). Overall, the mechanism usually requires a high concentration and sometimes external stimuli (temperature cycles, pH adjustment, ultrasonic, etc.) to reach a state where intermolecular attractions overcome thermal motion, yielding a percolating network of molecules. The specific roles of TCM components in hydrogel network formation are summarized in Supplementary Table 4.

### 4.2 Structural characteristics

Self-assembled TCM hydrogels are typically physical hydrogels, meaning no covalent bonds are formed during gelation (in contrast to chemical cross-linking). This imparts them with certain distinctive characteristics (Ding and Wang, 2017; Li et al., 2022). Microscopically, they often exhibit nanofiber or nanoribbon structures under electron microscopy (Zhao et al., 2024c). For instance, mangiferin hydrogel shows a mesh of nanofibers which correlate with its ability to hold water and be injectable (Hao et al., 2024). Glycyrrhizin hydrogels might display a lamellar or fibrillar structure due to how saponin molecules stack (Uman et al., 2020). These hydrogels are generally softer (lower storage modulus) than heavily cross-linked polymer gels, but interestingly they can still be quite robust depending on the network densit (Xu et al., 2022). A notable property often seen is shear-thinning and self-healing behavior. Because the cross-links are non-covalent and reversible, applying shear stress (e.g., during injection through a syringe) can break the interactions and fluidize the gel, and when the stress is removed, the interactions re-form and the gel solidifies again (Uman et al., 2020). This was observed in the mangiferin hydrogel–it was injectable and self-healing–making it convenient for application into wounds (Hao et al., 2024). Similarly, a glycyrrhizic acid-based hydrogel was reported to have self-healing properties and tunable viscoelasticity by adjusting concentration (Ji et al., 2025). The mechanical strength of self-assembled small-molecule hydrogels is sometimes lower than polymeric ones, but double-network approaches or composite with polymers can improve it (Gong, 2010; Li et al., 2023a). Notably, high small-molecule content can achieve moderate stiffness; for instance, glycyrrhizic acid-based gels at 10%–20% w/v demonstrate storage moduli of 1–10 kPa (Li et al., 2023a). These approaches have yielded hydrogels with mechanical properties comparable to soft tissues, expanding their potential for biomedical applications (Means and Grunlan, 2019). Another feature is biodegradability: since these are typically made of natural metabolites or close derivatives, they can be enzymatically or hydrolytically broken down in the body. Glycyrrhizin can be metabolized by glucosidases to GA, mangiferin by glycosidases as well; thus the gel will gradually disassemble *in vivo*, leaving no persistent synthetic polymer residue (Hao et al., 2024). Water content in these gels is very high (often >90%) (Ho et al., 2022), like other hydrogels, which is beneficial for maintaining a moist wound interface (Li et al., 2014; Li et al., 2016b).

### 4.3 Biological functions

Perhaps the most compelling aspect of TCM self-assembled hydrogels is that the gelator itself is usually biologically active. This means the entire gel matrix is therapeutically “loaded” (Zhao et al., 2009; Zhi et al., 2020). For example, glycyrrhizin-based hydrogels inherently carry glycyrrhizin’s pharmacological effects: it is an anti-inflammatory agent (via HMGB1 inhibition) (Paudel et al., 2020; Umeoguaju et al., 2021) and has some antimicrobial and antioxidant properties, particularly against Gram-positive bacteria like *Staphylococcus aureus* (Zhao et al., 2020b; Li et al., 2023b). Mees et al. (2023) found that glycyrrhizin hydrogels significantly accelerated wound closure in both normal and diabetic mice, primarily by enhancing keratinocyte migration. This suggests that the presence of glycyrrhizin in the matrix was bioactive throughout the healing process, likely modulating inflammation and perhaps interacting with growth factor signaling (HMGB1 is implicated in prolonged inflammation and impaired repair). For mangiferin, the self-assembled hydrogel (MF-gel) was shown to promote multiple aspects of wound healing: it scavenged intracellular ROS *in vitro*, promoted endothelial cell angiogenesis, and in diabetic wound models it accelerated wound contraction, increased collagen deposition, and enhanced angiogenesis (Lwin et al., 2021; Wu et al., 2022c; Hao et al., 2024). All these effects can be attributed to mangiferin’s known bioactivities (antioxidant, pro-angiogenic, anti-inflammatory) being effectively delivered by the gel (Mankar et al., 2025). Furthermore, because no foreign carriers were used, there were no issues of carrier toxicity or interference (Hao et al., 2024). Another interesting biological feature of some self-assembled systems is selective antimicrobial activity. A recent study of a glycyrrhizic acid/puerarin co-assembly reported that the hydrogel showed selective antibacterial effects against certain bacteria, which might be due to the presence of glycyrrhetinic acid moieties known to disrupt microbial membranes (Ji et al., 2025). Also, asiaticoside (from *Centella asiatica*) is a triterpene glycoside that has been formulated into a self-assembling injectable hydrogel with glycyrrhizic acid for wound healing, where both components are active: asiaticoside stimulates collagen synthesis and glycyrrhizic acid reduces inflammation (Zhang et al., 2024b). It stimulates collagen synthesis, promotes fibroblast proliferation, and enhances extracellular matrix accumulation in experimental wounds (Yuliati et al., 2015; Thong-on et al., 2024). Self-assembled TCM hydrogels can also be designed to be multi-functional: for instance, loading an additional drug into the network can provide dual function–the gel itself modulates the wound and the loaded drug addresses infection. For instance, a glycyrrhizin-based hydrogel loaded with carvacrol showed enhanced antibacterial activity and promoted healing of infected wounds (Cui et al., 2024). Similarly, a glycyrrhizic acid hydrogel exhibited anti-inflammatory and antibacterial properties while facilitating wound healing (Wu et al., 2023a). Other studies have explored hydrogels based on cinnamaldehyde and folic acid, hyaluronic acid and glycyrrhizic acid (Cui et al., 2024), and puerarin and silk fibroin (Wang et al., 2025), all demonstrating multifunctional wound healing properties. The porous nanofiber networks often allow efficient loading of secondary molecules if needed (Sun et al., 2022; Yang et al., 2024a). Lastly, these gels often have excellent biocompatibility as they are composed of natural compounds; cytocompatibility assays usually show high cell viability with the gels, and degradation products are metabolites the body can handle.

**FIGURE 3:**
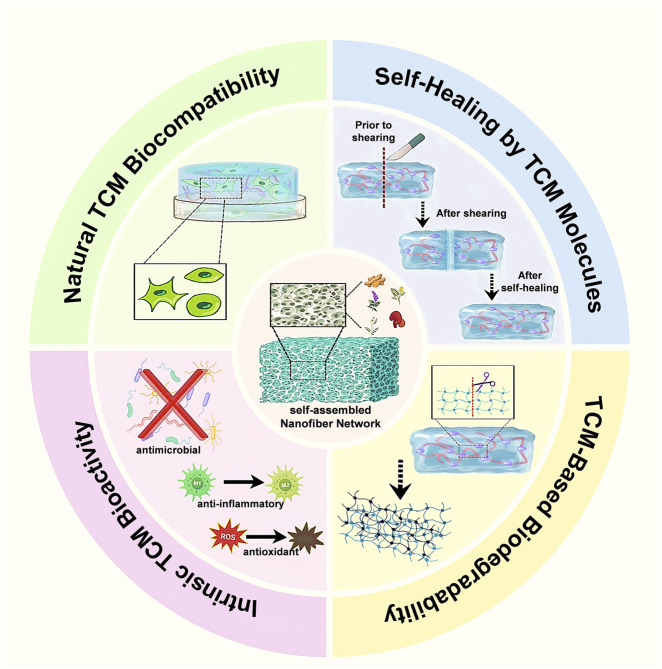
Material advantages endowed by self-assembled TCM nanofiber hydrogels. Four contiguous sectors emphasize key benefits: natural TCM biocompatibility that supports fibroblast adhesion and proliferation; intrinsic TCM bioactivity providing antimicrobial, anti-inflammatory, and antioxidant effects; TCM-based biodegradability permitting gradual hydrolytic disassembly into biotolerant fragments; and a self-healing property arising from reversible non-covalent interactions among TCM molecules, enabling rapid structural recovery after shear damage.

In summary, self-assembling hydrogels derived from TCM constituents, including glycyrrhizin, glycyrrhetinic acid, mangiferin, and related small molecules, represent an innovative class of biomaterials wherein the traditional boundary between active pharmaceutical ingredient and structural matrix becomes indistinct. Driven by supramolecular interactions, these molecules spontaneously form dynamic hydrogel networks characterized by intrinsic responsiveness, such as shear-thinning behavior and self-healing capability. With moderate mechanical strength and high-water content, these hydrogels closely mimic soft biological tissues, making them particularly suitable for wound healing applications. Biologically, these TCM-based hydrogels confer inherent therapeutic functionalities—including anti-inflammatory, antioxidant, and pro-regenerative effects—directly to the wound site, obviating the need for additional carrier substances. This carrier-free design paradigm is especially advantageous for clinical translation due to simplified formulations, fewer regulatory hurdles, and accelerated development pathways when the natural components already possess established safety profiles. Despite these promising attributes, challenges persist in achieving reproducible and stable gelation under physiological conditions, as well as in scaling up the production of structurally complex natural products. Nevertheless, continued interdisciplinary advancements at the intersection of natural product chemistry and materials science promise to yield additional TCM-derived self-assembling hydrogel systems, exemplifying an effective synergy between traditional medicine and cutting-edge biomaterial technologies for enhanced wound treatment (Figure 3). Material properties of self-assembled TCM hydrogels are provided in Supplementary Table 5.

## 5 Recent advances (2020–2025): comparative performance of TCM hydrogel systems

In the past 5 years, there has been a surge of research exploring various TCM-based hydrogels for diabetic wound healing. These studies include *in vitro* cell experiments, *in vivo* diabetic wound models (mostly rodent models, some large animal studies), and even preliminary clinical evaluations or case reports. This section synthesizes data from representative studies published between 2020 and 2025, highlighting performance differences among various hydrogel systems in wound healing outcomes and distinguishing features of their design. Key metrics compared include wound closure rates, healing duration, histological quality of repair (including re-epithelialization, collagen deposition, and angiogenesis), as well as anti-inflammatory effects and antimicrobial efficacy. Examination of these recent data provides insights into the most effective strategies and highlights existing knowledge gaps requiring further investigation (Supplementary Table 6).

### 5.1 Curcumin-loaded hydrogels

Multiple groups have reported curcumin-based hydrogels. Pham et al. (2019) demonstrated a chitosan/Pluronic hydrogel with curcumin that achieved >98% wound closure in a mouse model by 14 days, significantly better than untreated controls. In 2023, Zhao et al. (2023) GOHA-PBA/Cur hydrogel (curcumin in gelatin-HA PBA gel) showed enhanced healing in diabetic mice, with histology revealing reduced inflammation and increased vascularization. A 2022 study used a 3D-printed GelMA (gelatin methacrylate) hydrogel with curcumin and adipose stem cells, which expedited healing in diabetic wounds compared to GelMA alone (Xia et al., 2022). Fan et al. (2024) developed a PVA–chitosan–alginate hydrogel crosslinked via Michael addition with curcumin, demonstrating antioxidant, antimicrobial, anti-inflammatory, and angiogenic effects, significantly accelerating diabetic wound healing. Chopra et al. (2023) developed a chitosan–PVA–curcumin hydrogel film with sustained curcumin release, antimicrobial activity, and anti-inflammatory properties, suggesting strong potential for improved wound healing. Alghamdi et al. (2024) recently showed that a dermal-derived extracellular-matrix hydrogel loaded with curcumin accelerated >90% wound closure by day 14 in STZ-diabetic rats, while boosting collagen deposition, tensile strength and VEGF expression and simultaneously suppressing TNF-α/IL-1β-mediated inflammation. Generally, curcumin hydrogels stand out for their strong anti-inflammatory and antioxidant effect, often leading to faster resolution of the chronic inflammation phase. However, curcumin alone may not address severe infections, so some studies combined curcumin with antimicrobials or nanoparticles.

### 5.2 Baicalein/baicalin hydrogels

A notable example is the baicalein-loaded glycol chitosan/gellan gum hydrogel by Baviskar and Lodhi (2023). In streptozotocin-induced diabetic rats, wounds treated with this hydrogel healed completely by day 18, whereas control wounds were still not fully epithelialized. This baicalein hydrogel significantly increased antioxidant enzyme levels (SOD, CAT) in the wound and stimulated higher hydroxyproline content, indicating better collagen deposition. Another study by Zhu et al. (2024) investigated a *Scutellaria* extract (rich in baicalin and baicalein) encapsulated in a carboxymethyl chitosan/Sesbania gum hydrogel, demonstrating accelerated wound healing in diabetic mice infected with *S. aureus*. The hydrogel-treated wounds exhibited rapid re-epithelialization and organized collagen deposition, in contrast to persistent inflammation observed in untreated infected controls. Qin et al. (2025) developed an injectable photo-crosslinked GelMA nanocomposite hydrogel (BGZ@GelMA) co-loaded with baicalein, glucose oxidase, and ZIF-8. In streptozotocin-induced diabetic rats, wounds treated with this formulation healed significantly faster than controls. These results underline the potent wound-healing effect of baicalein/baicalin, delivered effectively via hydrogels, which improve the redox balance and inflammation in diabetic wounds.

### 5.3 Triterpenoid (GA) hydrogels

Mees et al. (2023) reported that a glycyrrhizin-based physical hydrogel enhanced epithelial coverage and accelerated wound closure in a diabetic mouse model by promoting keratinocyte migration. It’s noteworthy that even without an added drug, the glycyrrhizin gel itself served as the therapeutic. Separately, a supramolecular hydrogel derived from glycyrrhetinic acid (GA) (Guo et al., 2022) demonstrated potent antibacterial activity and accelerated wound healing in a rat skin defect model with bacterial infection. Additionally, Sun et al. (Zou et al., 2023) developed a sprayable GA hydrogel for postoperative adhesion (as previously mentioned); while that was for abdominal adhesion, they demonstrated in rats that GA hydrogel reduces fibrosis by releasing GA to inhibit inflammation. This concept could translate to cutaneous wounds, where a spray GA gel might reduce scar formation. Broadly, triterpenoid gels help by providing sustained anti-inflammatory action and in some cases antibacterial effects (GA is known to disrupt bacterial biofilms).

### 5.4 Polysaccharide-based hydrogels

The Ganoderma lucidum OGLP/CMC/SA hydrogel showed impressive results in diabetic wounds (Li et al., 2024a). In those wounds, the hydrogel significantly reduced M1 macrophages and increased M2, leading to faster granulation and skin appendage regeneration. Wounds treated with the GLP hydrogel had more mature collagen fibers and even hair follicle development, indicating a higher quality of healing. Another notable example is the Astragalus and Panax notoginseng extract hydrogel (APCS) developed in 2025, which significantly accelerated wound closure in rats, enhanced angiogenesis (evidenced by increased VEGF expression), and improved markers of nerve regeneration (elevated PGP9.5 expression) (Li et al., 2025b). This suggests that incorporating traditional multi-herb combinations into hydrogels can address multiple facets of wound repair–inflammation, blood flow, innervation. A purely polysaccharide-based material, the APS/CM-chitosan/alginate electroconductive hydrogelintegrated a conductive component to stimulate wound healing electrically. They reported not only improved wound healing but also better muscle tissue function around the wound–potentially significant for foot ulcers that can cause muscle atrophy (Tang et al., 2025). The conductivity (via added graphene or polypyrrole) plus APS’s bioactivity provided a unique synergy.

### 5.5 Composite multi-functional hydrogels

Recent studies often combine several elements: e.g., microporous hydrogels that deliver both herbal extracts and growth factors, or nanocomposite hydrogels with herbal drugs and inorganic nanoparticles. Zhang et al. (2023) engineered a pH-responsive hydrogel that sequentially releases Ag/curcumin-loaded polydopamine nanoparticles and VEGF, delivering antibacterial, anti-inflammatory, and pro-angiogenic effects that markedly hasten diabetic wound closure. Chen et al. (Shi et al., 2024) developed an injectable, ROS-responsive HA@Cur@Ag hydrogel (thiolated hyaluronic acid + hyperbranched PEG, loaded with curcumin liposomes and AgNPs). The material’s combined antioxidant, antibacterial, anti-inflammatory, and pro-angiogenic actions significantly accelerated diabetic wound closure *in vivo*. You et al. (Yan et al., 2023) encapsulated sericin-decorated curcumin and AgNPs within a silk-fibroin hydrogel, whose combined antibacterial, antioxidant, anti-inflammatory, and pro-angiogenic actions markedly accelerated diabetic wound healing. Likewise, hybrid hydrogels with stem cells and TCM compounds are emerging. Curcumin-loaded gelatin methacryloyl (GelMA) hydrogels have shown promise in reducing oxidative stress and improving adipose-derived stem cell survival in diabetic wounds (Xia et al., 2022). Similar hydrogels combining curcumin with chitosan and gelatin have demonstrated enhanced wound healing properties (Khoshmaram et al., 2024).

## 6 Mechanistic pathways of TCM components in diabetic wound healing

**FIGURE 4:**
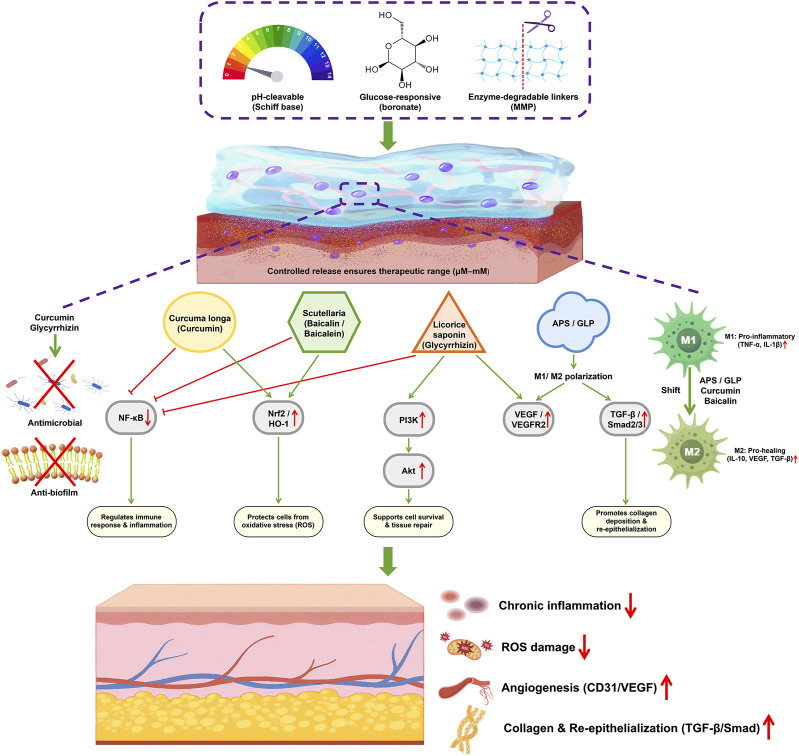
Mechanistic pathways of TCM-derived bioactive compounds in hydrogels for diabetic wound healing. TCM-derived hydrogels promote diabetic wound healing by targeting multiple pathways, including inhibition of NF-κB, activation of Nrf2/HO-1 and PI3K/Akt, and regulation of M1/M2 macrophage polarization, leading to reduced inflammation, decreased ROS, enhanced angiogenesis, and improved collagen deposition and re-epithelialization.

Elucidating the molecular mechanisms underlying the therapeutic efficacy of Traditional Chinese Medicine (TCM)-derived hydrogels is essential for their clinical translation. Diabetic wounds present complex pathological features, including dysregulated inflammation, excessive oxidative stress, impaired angiogenesis, and reduced cellular migration. This section systematically reviews the signaling pathways and molecular mechanisms modulated by major TCM-derived components—such as curcumin, baicalin, glycyrrhetinic acid, Astragalus polysaccharides (APS), and Ganoderma lucidum polysaccharides (GLP)—highlighting their roles in promoting diabetic wound repair (Figure 4). Clarifying these molecular mechanisms provides scientific validation for the therapeutic potential of these natural compounds and informs strategies for rational design and synergistic combination of bioactive hydrogels. A compound-to-pathogenesis mapping is further provided in Supplementary Table 7.

### 6.1 Curcumin-modulating NF-κB and Nrf2 signaling

Curcumin’s broad wound-healing activity is largely attributed to its dual role as an anti-inflammatory and antioxidant agent. On the inflammatory front, curcumin potently inhibits the NF-κB pathway, which is a central regulator of pro-inflammatory cytokine production. Curcumin suppresses activation of NF-κB by blocking the phosphorylation cascade (IKK, AKT/PI3K) that normally leads to NF-κB nuclear translocation (Kumari et al., 2022). As a result, curcumin-treated wounds show reduced levels of tumor necrosis factor-α (TNF-α), interleukin-1β (IL-1β), and other cytokines that drive chronic inflammation. It can directly interact with Keap1 (the Nrf2 inhibitor) via its electrophilic groups, leading to Nrf2 stabilization and nuclear translocation (Ramachandran et al., 2022). Consequently, curcumin elevates the expression of cytoprotective enzymes like heme oxygenase-1 (HO-1), superoxide dismutase (SOD), and glutathione peroxidase in wound tissues. In diabetic wound models, topical curcumin has been shown to increase Nrf2 and HO-1 levels while reducing ROS and malondialdehyde (MDA) accumulation, thereby mitigating oxidative damage (Wu et al., 2023b). This Nrf2-mediated antioxidant effect protects cells (keratinocytes, fibroblasts) from high-glucose-induced oxidative stress and creates a more favorable environment for healing. Beyond NF-κB and Nrf2, curcumin also influences other pathways: it binds to and inhibits COX-2, reducing prostaglandin synthesis; it activates PPAR-γ which helps resolve inflammation; and it can disrupt TLR4–MD2 receptor signaling, thereby lowering LPS-induced inflammatory responses (Zamanian et al., 2024). Collectively, through multi-pathway modulation (primarily NF-κB downregulation and Nrf2 upregulation), curcumin accelerates wound closure by dampening chronic inflammation, scavenging excess ROS, enhancing collagen deposition, and even promoting angiogenesis (via growth factor upregulation). In hydrogel formulations, these mechanisms are sustained over time, leading to faster resolution of the inflammatory phase and progression to the proliferative phase of healing.

### 6.2 Baicalein/baicalin–regulating macrophage polarization and redox balance

Baicalein and baicalin primarily aid wound healing by shifting the balance from a pro-inflammatory state to a pro-healing state at the wound site. A pivotal mechanism is their effect on macrophages–the key immune cells orchestrating wound repair. Diabetic wounds tend to have macrophages locked in a classically activated, pro-inflammatory M1 phenotype that overproduce cytokines and ROS. Baicalin has been shown to alleviate this inflammatory phenotype. In a diabetic mouse wound model, baicalin treatment resulted in significantly lower nuclear NF-κB levels in macrophages and higher cytoplasmic (inactive) NF-κB, indicating baicalin inhibits NF-κB activation (Yan et al., 2024). Concurrently, baicalin increased nuclear translocation of Nrf2 in those cells, enhancing antioxidant defenses. This leads to reduced intracellular ROS levels and downregulation of pro-inflammatory cytokines (IL-1β, IL-6, TNF-α) while upregulating anti-inflammatory cytokines like IL-10. As a result, baicalin-treated wounds show a promotion of M2 macrophage polarization–macrophages exhibit more Arg-1^+^/CD206^+^ (alternatively activated) phenotype and fewer iNOS^+^/CD80^+^ (M1 markers) compared to untreated wounds (Cai et al., 2021; Kim et al., 2023; Yan et al., 2024). Indeed, baicalin-treated wounds in diabetic mice had higher levels of TGF-β1 and IL-10, correlating with enhanced granulation tissue and faster re-epithelialization (Kim et al., 2023). Additionally, angiogenesis is improved: baicalin increased CD31^+^ microvessel density in wound tissue, likely via upregulating pro-angiogenic factors (VEGF, basic FGF) secondary to macrophage M2 activation. Baicalein’s direct antioxidant properties (as a polyphenol) also help restore redox balance–studies reported increased activities of SOD and catalase in baicalein-hydrogel treated wounds, indicating mitigation of oxidative stress. Furthermore, baicalein can inhibit inflammatory mediators like high mobility group box-1 (HMGB1) and STAT1, which are involved in macrophage M1 polarization (Baviskar and Lodhi, 2024). By lowering oxidative stress and inflammatory signaling, while boosting the regenerative, pro-collagen milieu (via TGF-β1 and other M2-related factors), baicalein/baicalin significantly accelerates diabetic wound closure. Encapsulation in hydrogels prolongs these effects and targets the delivery to wound tissues, as evidenced by complete healing observed with baicalein hydrogels in ∼2 to 3 weeks versus incomplete healing in controls. In summary, baicalein’s mechanisms center on reprogramming immune responses (M1→M2) and protecting tissue from oxidative damage, thereby breaking the cycle of chronic inflammation in diabetic wounds.

### 6.3 Glycyrrhetinic acid (GA) and glycyrrhizin–sustained anti-inflammatory and pro-regenerative actions

Licorice-derived triterpenoids GA and glycyrrhizin contribute to wound healing predominantly through anti-inflammatory mechanisms and support of tissue reconstruction. Glycyrrhizin is known to bind and inhibit HMGB1, a damage-associated molecular pattern that amplifies inflammation. In wound models, glycyrrhizin and GA consistently show downregulation of NF-κB signaling. For instance, in human keratinocytes, glycyrrhizin was found to reduce TNF-α-induced ICAM-1 expression via blocking the NF-κB/MAPK pathway (Leite et al., 2023). By interfering with upstream mediators (like IκB kinase and p38 MAPK), these compounds prevent NF-κB from inducing adhesion molecules and cytokines that recruit neutrophils and macrophages. The net effect is a temperance of the inflammatory response–wounds treated with glycyrrhizin showary tissue damage and allows the healing process to proceed (Fatima et al., 2024). Notably, glycyrrhizic acid has been reported to (particularly keratinocyte re-epithelialization) via NF-κB-dependent pathways: by partially inhibiting NF-κB, it seems to strike a balance that favors regeneration over inflammation (Mees et al., 2023). On the regenerative side, glycyrrhizin promotes angiogenesis and matrix remodeling. Treatment with glycyrrhizin-containing hydrogels led to upregulation of VEGF and miRNA-21 in wound tissue, correlating with increased capillary density and faster granulation (Hao et al., 2020). miR-21 is known to promote angiogenesis and collagen production, suggesting glycyrrhizin may exert gene regulatory effects that benefit healing (Xie et al., 2022). In addition, glycyrrhizin has been shown to inhibit NO synthase and reduce NO overproduction, which can otherwise delay healing by causing nitrosative stress (Dang et al., 2021). GA and glycyrrhizin also exhibit direct antimicrobial properties (especially against bacteria like *Staphylococcus*), helping to control infection in wounds (Al-Kubaise and Al-Shahwany, 2024). By providing sustained anti-inflammatory action (through NF-κB suppression and HMGB1 inhibition) and simultaneously supporting the proliferative phase (stimulating fibroblast proliferation, collagen deposition, and new blood vessel formation), licorice saponins create conditions for improved tissue regeneration (Richard, 2021). In diabetic rat wound models, a glycyrrhizin hydrogel significantly accelerated wound closure and epithelial coverage, even without any added antibiotic or growth factor, underlining that the compound itself was therapeutic. Overall, GA/glycyrrhizin serve as multifunctional agents that quell excessive inflammation while actively promoting the reconstructive aspects of healing (angiogenesis, epithelialization), making them especially valuable in chronic non-healing wounds (Qian et al., 2022).

### 6.4 Astragalus polysaccharides (APS) – immune regulation via Wnt/β-catenin and NF-κB

APS are prominent for their immunomodulatory capability, which is pivotal in healing chronic diabetic wounds. Recent mechanistic studies have elucidated that APS promotes wound healing largely by modulating macrophage activity through the β-catenin/NF-κB axis. APS was found to upregulate Wnt/β-catenin signaling (e.g., increasing β-catenin and its positive regulator R-spondin3) while concurrently inhibiting NF-κB and GSK-3β in wound macrophages. GSK-3β inhibition by APS further stabilizes β-catenin, creating a feed-forward enhancement of Wnt signaling. This molecular shift drives macrophages from the M1 state to the M2 state (Zhen et al., 2024). In diabetic ulcer models, APS treatment significantly reduced M1-associated inflammatory mediators and increased M2 markers, effectively reducing excessive inflammation in the late phase of wound healing. This M2 polarization is associated with higher secretion of TGF-β1, IL-10, and vascular endothelial growth factor (VEGF), which collectively improve granulation tissue formation and angiogenesis (Louiselle et al., 2021; Tu et al., 2021). Indeed, APS-treated wounds show faster resolution of inflammation and more robust granulation and revascularization than untreated diabetic wounds. Another study by indicated that APS can influence the PI3K/Akt/mTOR pathway: APS upregulated PTEN (a negative regulator of PI3K), thereby dampening overactive Akt/mTOR signaling in diabetic wounds. Overactivation of mTOR in diabetes can impair autophagy and cell survival; by moderating this pathway, APS helps restore a pro-healing cellular environment (Ma, 2022). In practical terms, APS’s mechanisms result in lower levels of neutrophil elastase and MMPs (due to reduced inflammation), improved collagen deposition (through TGF-β1 stimulation), and enhanced neoangiogenesis (through VEGF and IL-10 support) (Sha et al., 2023). APS also has intrinsic antioxidant effects, scavenging free radicals and upregulating antioxidant enzymes, which protect tissue in the hyperglycemic oxidative environment. By tackling inflammation and oxidative stress simultaneously, APS addresses two major impediments to diabetic wound healing (Zhen et al., 2024). This comprehensive mechanism-of-action explains the markedly improved healing (faster closure, better histological maturity) observed in APS-based hydrogel treatments of diabetic wounds.

### 6.5 Ganoderma polysaccharides–antioxidant and M2 polarization effects

GLP share some mechanistic similarities with APS, given both are immunomodulatory polysaccharides. GLPs are potent antioxidants and immunoregulators: they directly scavenge ROS and enhance the antioxidant defense system in chronic wounds (Zou et al., 2025). By reducing oxidative stress, GLP prevents further damage to cells at the wound margin and promotes a conducive environment for cell migration and proliferation. Mechanistically, GLP can activate Nrf2-mediated pathways, as suggested by increased expression of HO-1 and SOD in GLP-treated cells (Zhu et al., 2020). Additionally, GLP has been shown to modulate T cell and macrophage activity, skewing responses towards tissue repair. In diabetic wound healing studies, a GLP-containing hydrogel significantly decreased the proportion of M1 macrophages and increased M2 macrophages in the wound tissue (Ding et al., 2025). This aligns with observations that GLP can downregulate pro-inflammatory cytokines (e.g., TNF-α, IL-6) and upregulate anti-inflammatory IL-10. The enhanced M2 presence under GLP leads to earlier and richer granulation tissue and even stimulation of skin appendage regeneration (like hair follicles) in the healed wound (Mehdi et al., 2023; Wang et al., 2024). GLPs may exert these effects through toll-like receptor (TLR) signaling modulation; some studies indicate GLP can bind to immune cell receptors and trigger pathways (like MAPK and PI3K/Akt) that favor regenerative gene expression profiles (Lebrun et al., 2023). Another important aspect is angiogenesis: GLP treatment was associated with elevated VEGF in wound tissue and more robust neovascularization. This is partly due to M2 macrophages producing VEGF, and possibly GLP directly activating endothelial cells (Zou et al., 2025). Moreover, GLP’s antifibrotic property (observed in other contexts like liver fibrosis) might translate to improved collagen remodeling in wounds, preventing excessive scarring while ensuring tensile strength (Lindner et al., 2015). Summarizing, Ganoderma polysaccharides facilitate diabetic wound healing by quenching oxidative stress and reprogramming the immune response towards healing (increasing M2 macrophages), which results in accelerated inflammation resolution, timely angiogenesis, and quality tissue regeneration. Antimicrobial actions of TCM-derived hydrogels are summarized in Supplementary Table 8.

In essence, each TCM-derived component operates through one or more convergent pathways–curcumin and baicalein focus on NF-κB/Nrf2 (inflammation–oxidation axis); GA/glycyrrhizin on NF-κB and growth factor induction; APS and GLP on immune modulation via NF-κB inhibition and pro-healing macrophage activation. These mechanisms complement each other, which is why combining multiple TCM ingredients in hydrogels (or pairing with conventional therapies) can have additive or synergistic effects.

In summary, although these compounds may participate in hydrogel network stabilization through hydrogen bonding, π–π stacking, or Schiff-base linkages, numerous studies have confirmed that their pharmacological activities are not lost. On the contrary, they are preserved or even enhanced due to improved stability and sustained release (Mees et al., 2023; Baviskar and Lodhi, 2023). Therefore, hydrogels serve not only as drug carriers but also as amplifiers of the therapeutic potential of TCM-derived active compounds.

## 7 Challenges and limitations

Despite the promising advances of TCM-derived hydrogels for diabetic wound healing, several challenges and limitations remain before successful clinical translation can be achieved. First, variability in the purity and composition of bioactive compounds extracted from TCM herbs leads to inconsistent therapeutic outcomes and complicates reproducibility across studies. Second, most current research remains at the *in vitro* or small-animal stage, with a lack of large-scale, rigorously designed clinical trials to validate safety and efficacy in human patients. Third, pharmacokinetic and pharmacodynamic profiles of TCM-derived compounds within hydrogel matrices are not fully understood, particularly regarding their stability, release kinetics, and systemic absorption.

From a translational perspective, regulatory hurdles also pose a barrier, as TCM-based products often fall between conventional drug and biomaterial classifications, making approval pathways unclear. In addition, challenges in large-scale manufacturing, such as maintaining compound stability during hydrogel processing and storage, limit scalability for clinical application. Finally, batch-to-batch variability and the complex interaction between hydrogel carriers and active TCM compounds require further systematic study.

Addressing these challenges will require interdisciplinary efforts, including standardized extraction and characterization protocols, well-designed preclinical and clinical studies, and the development of scalable hydrogel fabrication techniques. Overcoming these hurdles could accelerate the clinical adoption of TCM-based hydrogels as multifunctional wound dressings in diabetic patients.

## 8 Conclusion

TCM-derived hydrogels represent an emerging class of advanced biomaterials capable of addressing the complex pathophysiology of diabetic wounds–including chronic inflammation, microbial infection, oxidative stress, and impaired tissue regeneration. By integrating bioactive TCM constituents such as curcumin, baicalein, glycyrrhetinic acid, and polysaccharides into hydrogel networks, significant therapeutic advantages have been demonstrated. These hybrid systems not only provide the traditional benefits of hydrogels (moisture retention, biocompatible coverage) but also actively modulate the wound microenvironment. For example, TCM-loaded hydrogels can release therapeutic agents in response to wound stimuli–altered pH, heightened enzymatic activity, temperature changes, or high glucose–thus delivering drugs “on demand” during infection or inflammation. They offer multifunctional effects: promoting rapid wound closure, enhancing granulation tissue and angiogenesis, and exerting antimicrobial and anti-inflammatory actions. Mechanistically, these hydrogels work by intervening in key molecular pathways (reducing NF-κB-mediated inflammation, boosting Nrf2/HO-1 antioxidative responses, stimulating PI3K/Akt and TGF-β/Smad for tissue repair, etc.), thereby tackling the root causes of impaired healing.

By bridging traditional herbal medicine with state-of-the-art biomaterials, TCM-based hydrogels embody a novel integrative strategy. They highlight how combining the multi-component, multi-target efficacy of TCM with modern delivery technologies can yield superior outcomes in regenerative therapy. Indeed, in preclinical studies these systems have significantly outperformed conventional wound dressings, and they align well with precision medicine approaches by tailoring treatment to the wound’s dynamic condition. Continued interdisciplinary research–involving natural product chemistry, bioengineering, and clinical trials–will be vital to further clarify modes of action, optimize formulations, and ensure safety and efficacy in human patients. In summary, TCM-derived hydrogels hold great promise as next-generation wound dressings that unify the wisdom of traditional medicine with the innovations of bioengineering to more effectively heal diabetic wounds and potentially other chronic injuries.
